# Buttoned Shut: Management of Complete Unilateral Nasal Obstruction Secondary to Button Battery Injury

**DOI:** 10.7759/cureus.37901

**Published:** 2023-04-20

**Authors:** Chloe B Harrington, Colin Bohr, Brian K Reilly, Michael Boyajian

**Affiliations:** 1 Otolaryngology, George Washington University School of Medicine and Health Sciences, Washington, D.C., USA; 2 Pediatric Otolaryngology, Children's National Hospital, Washington, D.C., USA; 3 Pediatric Plastic Surgery, Children's National Hospital, Washington, D.C., USA

**Keywords:** button battery injury, nasal obstruction, nasal reconstruction, pediatric facial plastic surgery, pediatric otolaryngology, nasal stenosis, reconstructive surgery

## Abstract

Pediatric button battery ingestion is known to cause potentially devastating injuries to the aerodigestive tract. Placement of a button battery in the nasal passages and subsequent damage it may cause poses a unique management problem as it may involve bony and membranous scarring, aesthetic irregularities, and long-term nasal obstruction. We present a case of a child with complete stenosis of the right nasal vestibule after a button battery injury. With a multidisciplinary surgical approach between an otolaryngologist and a plastic surgeon, the nasal airway patency was restored via a series of dilations and stents. The patient now has a patent right nasal airway that measures equal in diameter to the contralateral side. We conclude that in the case of a child with a button battery in the nose, repair of stenosis may be approached similarly to a case of unilateral choanal atresia, including dilations and stents.

## Introduction

Button batteries can cause long-term and potentially devastating injuries to the aerodigestive tract [1]. Management of these injuries includes identification and intervention as early as possible, either medically or surgically [2]. Placing a button battery in the nasal passage may have long-term consequences such as nasal stenosis, septal perforation, and structural deformities such as a saddle nose, which may be difficult to manage [3]. We present a case of a child who presented for care after a button battery injury was sustained to the right nasal vestibule.

## Case presentation

**Figure 1:**
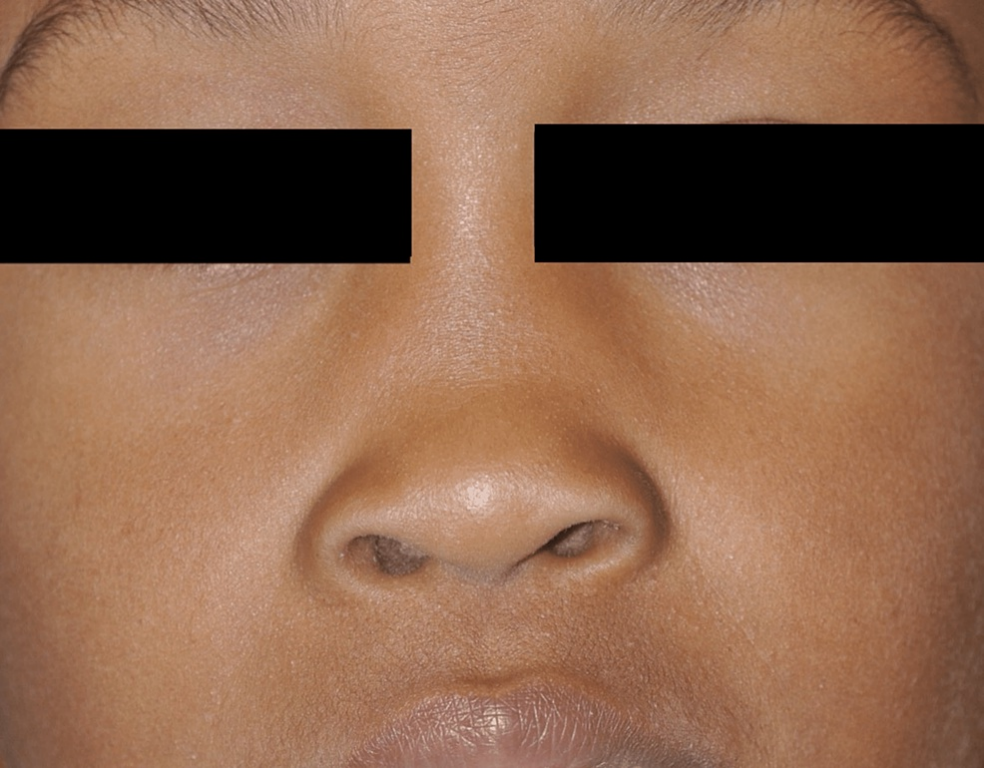
Photograph of the patient demonstrating right-sided saddle nose deformity.

**Figure 2:**
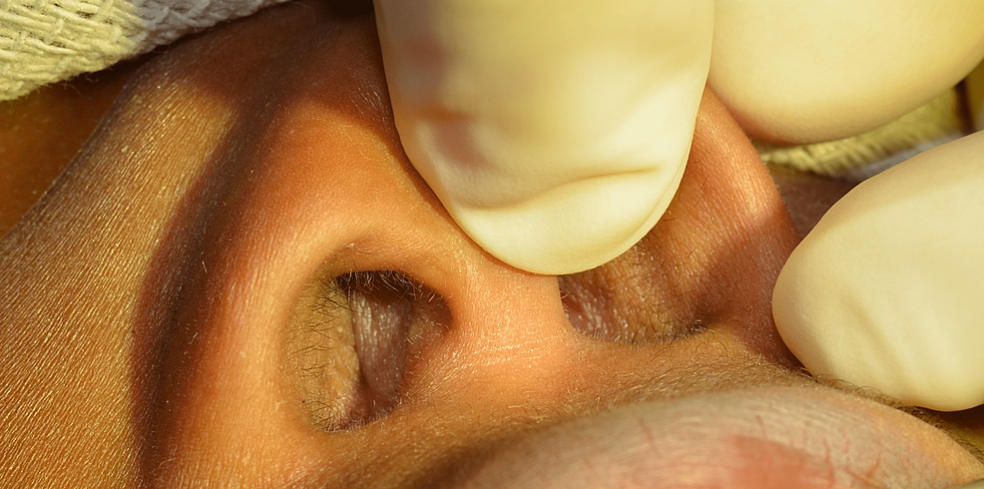
Photograph demonstrating complete obstruction of the right nasal cavity on anterior rhinoscopy.

We present a case of a child with complete stenosis of the right nasal vestibule after a button battery injury. He initially presented to our clinic for otolaryngologic evaluation at three years of age with a chief complaint of nasal obstruction and snoring. Six months prior to our evaluation, the patient reportedly inserted a button battery in the right nasal cavity, which remained unnoticed for three days before removal. Notably, the patient was living abroad when the foreign body was placed into the nose and those records were not available to us. On examination, he was found to have adenotonsillar hypertrophy and a right-sided saddle nose deformity (Figure 1). Anterior rhinoscopy showed complete obstruction of the right nasal airway with a well-mucosalized webbing (Figure 2). There was no evidence of ulceration or granulation tissue as the nasal cavity was completely obstructed by the scar tissue. The left nasal airway was normal and patent.

It was recommended that he undergo adenotonsillectomy with the concurrent repair of right nasal vestibular obstruction and the family was in agreement. He was referred to the plastic surgery department for further evaluation and reconstructive planning. Ultimately, the repair was deferred until the child was 10 years of age due to the fact that repair would require prolonged use of a nasal stent, which is often difficult for young children to tolerate and may jeopardize outcomes. Additionally, there were extenuating social circumstances for this particular child (he and his family moved overseas and back several times) and age 10 was the earliest appropriate opportunity for intervention. In the interim from our first evaluation until surgical repair, the patient was able to tolerate breathing through the left nasal passage and the mouth only.

**Figure 3:**
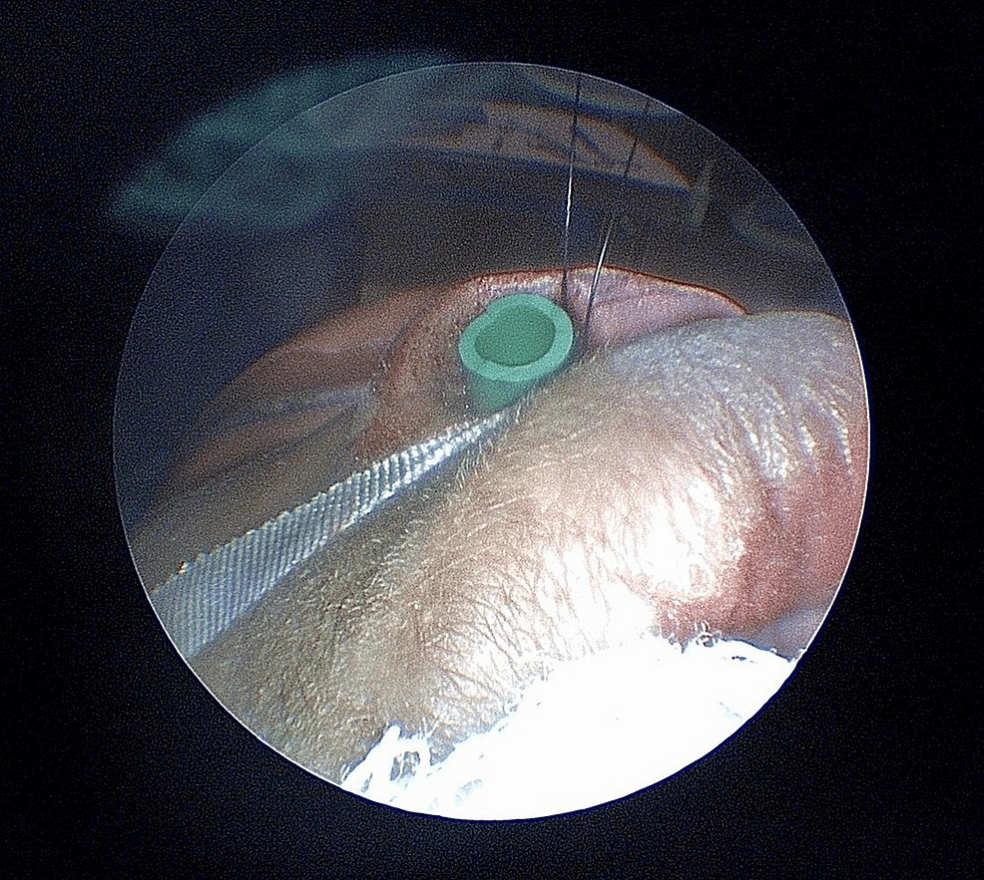
Intraoperative photograph of nasal stent fashioned from 24 French nasal airway being sutured into place.

The patient was taken to the operating room with the otolaryngology and plastic surgery teams. Adenotonsillectomy was performed at the start of the case with no complications. Endoscopy with a zero-degree endoscope was performed and revealed a complete obstruction of the right choana with fibrotic and bony components. Additionally noted was a septal perforation, which was likely caused by the initial button battery injury. The right nasal passage repair began with a three-limb incision into the mucosa covering the osseous components of the scar. Three mucosal flaps were elevated and the bony scar was removed using an osteotome and cutting rongeur. To prevent exposure of raw areas and to decrease the risk of re-scarring, the three flaps of mucosa were carefully laid in place in the newly reconstructed airway and sutured with 5-0 chromic. A silicone 14-French stent was shortened and sutured to the septum with a 3-0 nylon stitch (Figure 3).

The child returned for a follow-up examination in the clinic on postoperative day six and was found to be doing well with no discomfort caused by the stent. One month postoperatively, the child reported being able to breathe well through the right naris with complete resolution of his snoring and improvement in overall quality of sleep.

Approximately one and a half months after the initial surgery, the patient was brought back to the operating room for nasal endoscopy and stent upsizing. The nasal airway was noted to be patent but not equal in diameter to the contralateral side. There were mucosal adhesions present that were sharply released inferiorly. A long nasal speculum was used to further dilate the airway. A 24-French silastic tube was then easily inserted into the airway and sutured to the membranous septum with a 3-0 nylon stitch.

He was evaluated in the clinic two months after the stent upsize and the surgical site was noted to be healing well. He was breathing easily from his bilateral nasal passages and continued to be unbothered by the presence of the stent.

He underwent repeat endoscopic evaluation one month afterward (four months after the first surgery) at which time it was demonstrated that the right nasal passage was equivalent in diameter to the left, both easily permitting a 24 French nasal airway. This was sutured into place on the right and remained in place as a stent. This stent was left in place for approximately four months, at which time it was removed in the office and the child began a part-time home stenting regimen. For one month, the patient placed the stent in the nose at night and removed it in the morning but the patient's caregiver noted it was more difficult to replace after a few weeks. They attempted to keep the stent in place at all times but the patient experienced issues at school with bullying from other children regarding the stent. The current regimen consists of replacing the stent during all waking hours that the child is not at school, which has worked well for them. It has now been almost a year since surgical intervention first began and the plan is to continue the home stenting regimen. The patient’s saddle nose deformity does not cause the child any cosmetic concerns and there are no plans for surgical repair at this time, although the patient may elect for this in the future.

## Discussion

This case is an example of the long-lasting damage that button battery burns can inflict on the tissues of the aerodigestive tract. This is significant due to the increase in recent years of button battery-related emergency room visits [4]. A 2012 retrospective single-institution study demonstrated that over a 20-year period, 10.2% of all battery-related visits were due to nasal insertion [5]. This was the second most common chief complaint behind ingestion, which comprised 76.6% of these visits. Nasal insertion can present unique diagnostic challenges, especially if the foreign body is not suspected based on the initial history given to a clinician. They can remain in place for days before causing outward signs of a problem, often presenting with foul nasal discharge, pain, or swelling of the area.

The most important step in managing button batteries in the nose is to remove the battery as soon as possible. Septal perforation may occur in as little as three hours and inferior turbinate necrosis has occurred within 24 hours of button battery insertion [6,7]. Further management strategies primarily consist of addressing mucosal scarring as early as possible with topical treatments such as nasal saline and honey; however, by the time of ideal intervention, this patient had already developed significant osseous and fibrotic scarring [2].

In this case, due to delayed ability to provide care, the patient developed bony stenosis of the right nasal passage as well as a saddle nose deformity due to a nasal septal perforation sustained at the same time. The bony stenosis component in this case required a management approach similar to that of unilateral choanal atresia with revisions, destruction of bony scarring, and long-term stenting required in much the same way. Our unique case is an example of a button battery injury to the nasal cavity that was not able to be immediately addressed, therefore creating a more complex surgical problem.

## Conclusions

Button battery ingestion is generally caught early enough to intervene and prevent further damage. In this particular case, the ingestion was caught early, but the surgical intervention was delayed. This was partly by intention and partially due to social circumstances; however, an ideal outcome was achieved. Unfortunately, the delay allowed osseous scar formation that mandated an approach similar to choanal atresia repair. In that same vein, repeated scar lysis and stenting have proven to be successful in this patient. Delaying surgical intervention has been advantageous in that the patient is mature enough to tolerate intermittent stenting at home to maintain the equal diameter of the bilateral nares. In conclusion, there were both drawbacks and benefits to delaying surgical repair but ultimately there was a good end result for this patient.
